# Clinical efficacy of bone transport technology in Chinese older patients with infectious bone nonunion after open tibial fracture

**DOI:** 10.1186/s12877-021-02409-1

**Published:** 2021-09-07

**Authors:** Ying Wen, Peiming Liu, Zhichao Wang, Ning Li

**Affiliations:** Orthopedics Department, Harbin No. 5 Hospital, Harbin, 150040 China

**Keywords:** Bone transport technology, Bone nonunion, Open tibial fracture

## Abstract

**Objective:**

This study was designed for the first time to analyze clinical efficacy of bone transport technology in Chinese older patients with infectious bone nonunion after open tibial fracture.

**Methods:**

This study retrospectively analyzed 220 older patients with infectious bone nonunion after open tibial fracture. There were 110 patients receiving bone transport technology (Group A) and 110 patients receiving membrane induction technique with antibiotic bone cement (Group B).

**Results:**

There were 164 male patients and 56 female patients, with an age range of 65 to 71 years and an average age of 67 ± 1.3 years. Traffic accident, high-fall injury and crush injury account for 45.5, 27.7 and 26.8%, respectively. Age, gender, histories, causes and fracture location had no significant difference between the two groups (*P* > 0.05 for all). Operation time in the Group A was significantly shorter than that in the Group B (*P* < 0.05). Linear and positional alignment (70.9 vs. 57.3), American Knee Society knee function score (167.7 ± 14.9 vs. 123.8 ± 15.7), Baird-Jackson ankle function score (89.9 ± 3.5 vs. 78.4 ± 4.9), bone healing index (43.0 ± 2.0 vs. 44.3 ± 3.0) and clinical recovery (8.2 vs. 4.5) of patients in the Group A were significantly better than those in the Group B (*P* < 0.05 for all). Wound infection in the Group A (7.3%) was significantly less than that in the Group B (16.4%; *P* < 0.05). There were neither a neurovascular complication nor a recurrence of infection in the two groups.

**Conclusion:**

Bone transport technology achieved better knee and ankle joint function recovery and superior bone healing and clinical efficacy than membrane induction technique with antibiotic bone cement, suggesting that bone transport technique is worthy of extensive promotion to improve clinical condition of older patients with infectious bone nonunion after open tibial fracture.

## Introduction

With the development of society and the acceleration of industrialization, there are an increasing number of traffic and engineering accidents, and more cases of high-energy injuries and limb fractures [1]. Because patients are prone to have severe wound infection, defective skin necrosis and long-term wound healing after open fracture, it is very easy for patients to develop infectious bone nonunion in clinical practice, and open tibial fracture is one of the most common cause of infectious bone nonunion [2, 3]. There is weak tissue around the tibia, and infection is easy to happen after fracture. Infection after open tibial fracture is characterized by prolonged disease course, permanent functional deficits and high reinfection rates, which not only seriously affects life quality of patients, but also causes heavy burden on families and society [4]. Meanwhile, open tibial fracture is often accompanied by delayed and poor healing, and infectious bone nonunion is a more difficult problem in clinical treatment [5]. Tibial infectious nonunion is usually associated with bone deformities, bone loss, persistent infection and adverse effects on life quality [6]. Because older patients have low immune function and slow bone growth, they are more likely to have infectious bone nonunion after open tibial fracture. There were high therapeutic difficulty and poor therapeutic effects in older patients with infectious bone nonunion, posing a severe challenge for orthopedic surgeons and patients [7].

Clinical doctors urgently need to find an effective treatment with obvious efficacy in older patients with infectious bone nonunion after open tibial fracture [8]. Operative surgery is the main clinical treatment of infectious bone nonunion. Its principle is to promote bone healing by clearing infective lesions and killing necrotic tissues. However, traditional treatment is to resect injured tissues, prevent active infection and repair bone defect by free bone transplantation [9]. With the continuous improvement of medical level, bone transport technology has been applied by clinicians in the treatment of infectious bone nonunion. Based on the tension-stress law, bone transport technology might be promote bone regeneration and bone healing by achieving external fixation of infectious bone nonunion [10]. It has the potential to become significant method for infectious bone nonunion after open tibial fracture [11]. However, although several studies showed high successful rates with surgical treatment of infectious bone nonunion after open tibial fracture, the application of bone transport technology is still in the exploratory stage in the treatment of older patients with infectious bone nonunion after open tibial fracture [12–14]. General speaking, young patients are more likely to choose operative reconstruction, while older patients are more likely to choose operative amputation. However, amputation significantly reduces physical and mental well-being of older patients, and successful operative reconstruction could be a better alternative option [7]. Clinical efficacy of bone transport technology needs to be further studied in older patients with infectious bone nonunion after open tibial fracture. Therefore, the current study was designed for the first time to analyze clinical efficacy of bone transport technology in Chinese older patients with infectious bone nonunion after open tibial fracture.

## Methods

### Study participants

The current study retrospectively analyzed 220 older patients with infectious bone nonunion after open tibial fracture. All patients received operative treatment in Orthopedics Department, Harbin No. 5 Hospital, between July 2012 and November 2019. There were 110 patients receiving bone transport technology and allocated to Group A, and 110 patients receiving membrane induction technique with antibiotic bone cement and allocated to Group B. Inclusion criteria: 1) ≥ 65 years old; 2) open tibial fracture; 3) fracture-related infection: abscess, draining sinus, intraoperative purulence, significant growth of a microorganism from intraoperative tissue specimens (at least two or more positive sterile site cultures), and positive histopathology supportive of deep active infection; 4) interrupted bone healing with no sign of healing in the normal time. No bone healing after increasing treatment time and treatment was essential to achieve bone healing; and 5) signed informed consent, had complete data and were followed up. Exclusion criteria: 1) histories of studied treatments; 2) intraarticular or pathological fractures; 3) fractures with vascular compromise; and 4) malignant tumors or other reasons making patients not undergo operative surgery. The current study was in accordance with the Declaration of Helsinki and approved by the Ethics Committee of Harbin No. 5 Hospital (EC-20190730-1243), with the signed informed consent of all patients.

### Surgical procedures

Patients avoided all surgical treatment during the acute attack period and received surgical treatment after infection was controlled with antibiotics. Patient was placed in a supine position, with the affected limb raised and infected area exposed. Epidural anesthesia, strict disinfection and aseptic operation were conducted in surgical treatment when patients was stable and surgery was possible. Bone transport technology was conducted in patients (Group A) based on the following surgical procedures: 1) to resect infected, necrotic and scar tissues, protect significant nervous and vascular structures, peel off hardened and necrotic bones, and debride wound surface and necrotic space; 2) to fix ring external fixator in the metaphyseal of proximal and distal tibia, parallel to the upper and lower tibia rings and across osteotomy plane; 3) osteotomy was conducted at the proximal tibia with its plane horizontal with tibial nodule surface. 4) to maintain original length and force line of affected limb, and adjust linear and positional alignment; 5) to tighten the nut after all steel needles were fixed; 5) to suture whole wound and elevate affected limb; 6) bone transport was started within 7 days after the operation, and conducted by 0.25 mm/time, 4 times/d and 1 mm/d; 7) dressing is changed once a week, and needles are disinfected daily with alcohol; and 8) to stop bone transport and remove external fixator when new callus bone was solid and bone mineralization was basically mature.

Patients (Group B) with soft tissue defect were first treated with lesion resection, dressing change and skin grafting. After soft tissue was adequately covered (3 months later), these patients were treated with membrane induction technique with antibiotic bone cement. Patients without soft tissue defect were directly treated with membrane induction technique with antibiotic bone cement: 1) to resect infected, necrotic, inflammatory granulation and fibrous scar tissues, protect significant nervous and vascular structures, peel off hardened and necrotic bones, and debride wound surface and necrotic space; 2) to fill necrotic space using antibiotic bone cement with appropriate size and additional vancomycin; 3) to fix external fixator and make fracture stable; 4) bone cement was removed and bone graft was implanted after membrane induction was formed (6 to 8 weeks later); and 5) external fixator was removed after bone healing. For two groups, antimicrobial treatment was adjusted based on the susceptibility of pathogens and continued for at least 6 weeks after operation.

### Postoperative follow-up

There were standardized recording of demographic data and procedure details collected by a multidisciplinary expert team. These experts were from Orthopedics Department, Anesthesiology Department, Geriatric Department, Endocrinology department and Cardiology Department. They were not masked to surgical procedures. Patients were followed up for 2 years after surgical operation. Linear and positional alignment was checked in two groups, and bone healing index was compared between two groups. Bone healing index was the time required for each 1 cm extension. Knee function was evaluated using the American Knee Society (AKS) knee function score (200 points), including Knee score and Knee function score, each with 100 points [15]. Knee score included pain, motion and stability. Knee function score included the abilities to walk and climb stairs. AKS knee function score could fully evaluate knee anatomical and functional status, and the higher the score, the better the recovery of knee function. Ankle function was evaluated using the Baird-Jackson ankle function score (100 points) similar to AKS knee function score, and the higher the score, the better the recovery of ankle function [16]. Clinical efficacy was evaluated with the following criteria generally applied in clinical work: 1) complete recovery: complete healed limb, complete recovered function, movement without obstacle, complete physical self-maintenance and complete physical activity; 2) good recovery: good healing limb, good functional recovery, movement without obstacle, good physical self-maintenance and general physical activity; 3) moderate recovery: moderate healing limb, moderate functional recovery, movement with slight obstacle, moderate physical self-maintenance and losing physical activity; 4) poor recovery: poor healing limb, poor functional recovery, movement with obstacle, losing physical self-maintenance and losing physical activity.

### Statistical analyses

Continuous data with normal distribution were expressed as mean ± standard deviation, and Student’s t-test was used for comparison between groups. Continuous data with skewed distribution were expressed as medians (interquartile ranges), and Mann-Whitney U test was used for comparison between groups. Categorical data were expressed as numbers and percentages, and *χ*^*2*^ tests were used for comparison between groups. Statistic Package for Social Science 20.0 software package (SPSS Inc., Chicago, IL) was used for statistical analyses, with two-tailed *P* < 0.05 considered statistically significant in all tests.

## Results

There were 164 male patients and 56 female patients, with an age range of 65 to 71 years and an average age of 67 ± 1.3 years. Traffic accident, high-fall injury and crush injury account for 45.5, 27.7 and 26.8%, respectively. As shown in Table 1, age, gender, histories, causes and fracture location had no significant difference between the two groups (*P* > 0.05 for all). Operation time in the Group A was significantly shorter than that in the Group B (*P* < 0.05).

**Table 1:** Comparison of patient characteristics between groups before treatment

Item	Total (***n*** = 220)	Group A (***n*** = 110)	Group B (***n*** = 110)	***P*** value
Age (years, $\overline{x}$ ±s)	67 ± 1.3	67 ± 1.4	67 ± 1.2	0.651
Male [n (%)]	164 (74.5)	85 (77.3)	79 (71.8)	0.353
Histories [n (%)]				
Hypertension	137 (62.3)	71 (64.5)	66 (60.0)	0.487
Diabetes mellitus	65 (29.5)	34 (30.9)	31 (28.2)	0.658
Causes [n (%)]				0.737
Traffic accident	100 (45.5)	51 (46.4)	49 (44.5)	
High-fall injury	61 (27.7)	32 (29.1)	29 (26.4)	
Crush injury	59 (26.8)	27 (24.5)	32 (29.1)	
Fracture position [n (%)]				0.497
Left side	123 (55.9)	59 (53.6)	64 (58.2)	
Right side	97 (44.1)	51 (46.4)	46 (41.8)	
Operation time (min, $\overline{x}$ ±s)	144 ± 16.1	138 ± 17.2	150 ± 12.6	< 0.001

During a follow-up of 2 years, all patients had a follow-up rate of 100%. As shown in Table 2, linear and positional alignment (70.9 vs. 57.3), American Knee Society knee function score (167.7 ± 14.9 vs. 123.8 ± 15.7), Baird-Jackson ankle function score (89.9 ± 3.5 vs. 78.4 ± 4.9), bone healing index (43.0 ± 2.0 vs. 44.3 ± 3.0) and clinical recovery (8.2 vs. 4.5) of patients in the Group A were significantly better than those in the Group B (*P* < 0.05 for all). Wound infection in the Group A (7.3%) was significantly less than that in the Group B (16.4%; *P* < 0.05). There were neither a neurovascular complication nor a recurrence of infection in the two groups.

**Table 2:** Comparison of clinical efficacy between groups at 2 years after treatment

Characteristics	Total (***n*** = 220)	Group A (***n*** = 110)	Group B (***n*** = 110)	***P*** value
Counterpoint and alignment [n (%)]				0.035
Good	141 (64.1)	78 (70.9)	63 (57.3)	
Poor	79 (35.9)	32 (29.1)	47 (42.7)	
American Knee Society (score, $\overline{x}$ ±s)	145.8 ± 26.8	167.7 ± 14.9	123.8 ± 15.7	< 0.001
Baird-Jackson (score, $\overline{x}$ ±s)	84.1 ± 7.1	89.9 ± 3.5	78.4 ± 4.9	< 0.001
Bone healing index (d/cm, $\overline{x}$ ±s)	43.7 ± 2.6	43.0 ± 2.0	44.3 ± 3.0	< 0.001
Clinical efficacy [n (%)]				0.036
Complete recovery	14 (6.4)	9 (8.2)	5 (4.5)	
Good recovery	116 (52.7)	65 (59.1)	51 (46.4)	
Moderate recovery	62 (28.2)	28 (25.5)	34 (30.9)	
Poor recovery	28 (12.7)	8 (7.3)	20 (18.2)	
Complications [n (%)]				
Wound infection	26 (11.8)	8 (7.3)	18 (16.4)	0.022
Inequality of lower limb	11 (5.0)	4 (3.6)	7 (6.4)	0.353
Anchylosis	9 (4.1)	3 (2.7)	6 (5.5)	0.496

## Discussion

Infectious bone nonunion is difficult to treat and easy to recur in clinical practice. It is often caused by trauma and mostly occurs in the tibia [5]. Infectious bone nonunion caused by open tibial fracture has significantly affected physical and psychological health of patients, and obviously increased the burden of family and society [17]. Although there has been an improvement in the treatment of open tibial fractures since the advanced implantation and less traumatic surgical techniques, the infection rate is still high, ranging from 9 to 18% of patients, and its recurrence rate is very common, affecting 10 to 20% of patients [5]. The treatment of infectious bone nonunion is especially challenging in older patients with open tibial fracture. As the best treatment option of infectious bone nonunion after open tibial fracture, surgical operations include the following methods: 1) lesion removal, space filling, internal fixation and bone grafting; 2) membrane induction technique with antibiotic bone cement; and 3) bone transport technology. Lesion removal, space filling, internal fixation and bone grafting are traditionally and widely used operative methods for infectious bone nonunion after open tibial fracture. It improves patient’s condition through the thorough removal of inactivated bone and related soft tissue. However, it generally has high preoperative requirement for soft tissue and easily result in postoperative infection and bone nonunion. In the early stage after surgical operation, the affected limb can not bear certain weight, thus aggravating its osteoporosis in older patients.

Membrane induction technique with antibiotic bone cement has been proposed as a new strategy for the treatment of infectious bone nonunion after open tibial fracture [18]. It provides key physical and biological effects through inducing membrane formation. This membrane has similar structure and content of growth factor with autologous periosteum. With a thickness of 0.5-2 mm, it can secrete a variety of osteogenic precursor cells, osteogenic growth factors and vascular growth factors. Cortical bone defects were repaired by inducing rapid osteogenesis and cortical shaping of intramembranous cancellous bone. Meanwhile, implanted autologous cancellous bone itself has good osteoinductive and osteogenic properties. However, in addition to relatively long treatment time, membrane induction technique with antibiotic bone cement has high preoperative requirement for soft tissue, and causes certain trauma through extracting autologous ilium [5]. Although autologous bone is the most ideal source of implanted bone, lacking autologous bone limits the application of membrane induction technique.

Bone transport technique is a new technique to achieve bone and soft tissue repair through increasing fixation strength with stable three-dimensional fixation. Its basic principle is the tension-stress law of tensile tissue regeneration, that is, when biological tissues are slowly pulled to generate excellent axial stress stimulation, activate cell proliferation and vessel formation, and promote bone formation and healing [19, 20]. Bone transport technique firstly makes an appropriate osteotomy of bone stump, then uses an appropriate external fixator, and choose the appropriate time and speed to pull bone and soft tissues. Bone transport has the following advantages: 1) it has simple procedures, reliable clinical efficacy and shortened operative time; 2) it is a minimally invasive surgical technique that can correct deformities and shortening at the same process; 3) it simultaneously controls the infection and repair the defection of bone and soft tissues, especially applicable with poor soft tissue coverage; 4) there is no strict restriction on the length of defective bone, and it is needed to retain only one end of tibial metaphysis; 5) in the early stage after surgical operation, weight bearing on the affected limb reduces time in the bed, avoid continuous muscle wasting and avoid different perioperative complications.

Several published papers has discussed bone transport technique in treating infectious bone nonunion after open tibial fracture [12–14]. These studies have shown that bone transport technology could resect diseased and necrotic bone and pull bone and soft tissues to promote bone formation, correct the deformity and achieve functional recovery without bone grafting, However, the application of bone transport technology is still in the exploratory stage in the treatment of older patients with infectious bone nonunion after open tibial fracture. General speaking, young patients are more likely to choose operative reconstruction, while older patients are more likely to choose operative amputation. However, amputation significantly reduces physical and psychological well-being of older patients, and successful operative reconstruction could be a better alternative option [7]. Clinical efficacy of bone transport technology needs to be further studied in older patients with infectious bone nonunion after open tibial fracture. The current study demonstrated that bone transport technique not only takes less time, but also promote patients’ recovery than membrane induction technique with antibiotic bone cement. Linear and positional alignment, functional scores, bone healing and clinical efficacy of patients achieved by bone transport technique were significantly better than membrane induction technique with antibiotic bone cement, suggesting that bone transport technique is suitable for Chinese older patients with infectious bone nonunion after open tibial fracture.

Complete resection and debridement of necrotic tissue, long course of antimicrobial and supportive therapy and adequate reconstruction of bone and soft tissues, are of immense significance to effective achievement of bone transport technique in treating infectious bone nonunion after open tibial fracture [21, 22]. Due to rich blood supply, large bone surface and highly prevalent osteogenesis, tibial metaphysis is generally chosen as the osteotomy site to achieve rapid bone healing and good clinical efficacy [21]. Debriding wound and anti-infection treatment are essential to avoid new bone infection and infectious bone nonunion after open tibial fracture. The osteotomy site should meet the requirements of bone length, blood supply and periosteum completeness. Time and speed of bone transport are also significant to its clinical efficacy and can be determined based on soft tissue. Due to poor soft tissue coverage, bone transport might affect bone healing. If there is good soft tissue coverage, bone transport can be performed about 7 days after osteotomy and at the speed of 1 mm/d. Bone transport speed is mainly determined on clinical efficacy and can be slowed down in older patients. Bone transport direction can be appropriately adjusted based on bone healing, and older patients should be encouraged to bear proper weight after operation.

The current study had one limitation. The current study was a retrospective study, and further prospective study is essential to confirm our results. All researchers in the current study were not masked to surgical procedures, and randomized controlled trial with blind method should be performed to avoid the limitation.

## Conclusion

The current study demonstrated that bone transport technology achieved better knee and ankle joint function recovery and superior bone healing and clinical efficacy than membrane induction technique with antibiotic bone cement, suggesting that bone transport technique is worthy of extensive promotion to improve clinical condition of older patients with infectious bone nonunion after open tibial fracture.

## Data Availability

In attempt to preserve the privacy of patients, clinical data of patients will not be shared; data can be available from authors upon request.
